# Effect of the mixed ownership reform on the stock market: Evidence from China

**DOI:** 10.1371/journal.pone.0317927

**Published:** 2025-03-31

**Authors:** Xi Gu, Sijia Qiao, Yishi Wang, Tianqi Song

**Affiliations:** 1 College of Economics and Management, Shanghai Maritime University, Shanghai, Pudong District, China,; 2 Shanghai National Accounting Institute, Qingpu District, Shanghai, China,; 3 Shanghai Institute of Aerospace System Engineering, Minhang District, Shanghai, China; Open Polytechnic of New Zealand, NEW ZEALAND

## Abstract

This study examines the effect of the mixed ownership reform from the perspective of a capital market. Based on a comprehensive dataset of Chinese state-owned enterprises (SOEs) during the period of 2006–2020, this study determines that the mixed ownership reform can decrease corporate stock price synchronicity amongst SOEs, enhancing their capital allocation efficiency. The mediation analysis reveals that the mixed ownership reform restrains the stock price synchronicity of firms by enhancing executive incentive, strengthening checks and balances amongst shareholders and reducing administration constraints in SOEs. Additional tests demonstrate that this effect is more pronounced amongst firms whose chief executive officers wield greater authority, firms whose cash flow and control rights are separated and firms with higher marketisation level. Our study adds to the literature on the governance role of the mixed ownership reform by providing evidence at the capital market level.

## 1. Introduction

Corporate governance issues in state-owned enterprises (SOEs) have long been a concern amongst researchers worldwide. One of the issues is that SOEs are inevitably facing challenges, such as conflicting shareholder objectives, the absence of incentive mechanisms and corruption, due to their special capital structure; these challenges will hamper the effectiveness of SOEs (Laffont and Tirole, 1991; Qian, 1996; Shleifer and Vishny, 1994; Wei *et al*., 2017; Apriliyanti *et al*., 2024; Jia *et al*., 2024; Bae *et al*., 2024) [1–7]. To solve these problems, privatisation has become a viable solution in practice. Research and industry practices have provided evidence that privatising SOEs can optimise corporate governance, resulting in improved profitability, real sales and operational efficiency (Megginson *et al*., 1994; Boubakri and Cosset, 1998; Fink Matto and Rathindran, 2002) [8–10]. However, privatisation in SOEs may lead to undesirable rent extraction, triggering the loss of state assets (Filatotchev *et al*., 2003) [11]. Moreover, reform practices, such as shock therapy in the Soviet Union, have demonstrated that privatisation in SOEs cannot solve the aforementioned problems.

China has implemented a unique scheme for reforming its SOEs. Instead of privatisation, the Chinese government has implemented the ‘mixed ownership reform’ to enhance corporate governance in SOEs. The mixed ownership reform attempts to introduce non-state-owned capital into SOEs. By working with one another, different types of shareholders exert their advantage when participating in business decision-making and corporate governance, promoting the reform and reshaping of institutional mechanisms, such as corporate governance, state-owned asset operation supervision and profit distribution (Yan *et al*., 2023; Gu and Jia, 2022) [12,13]. In particular, SOEs have several methods for implementing the mixed ownership reform, including increased capital and shares, bid for listing, equity transfer and employee stock ownership. Through these methods, different types of shareholders are introduced into SOEs and engaged in their management and operation. Previous studies have mostly investigated the governance effect of the mixed ownership reform from the firm level, including how the mixed ownership reform affects the governance structure of SOEs (Boateng and Huang, 2016), capital allocation efficiency (Guan *et al*., 2021), tax avoidance (Wang *et al*., 2021), corporate innovation (Li *et al*., 2019) and corporate social responsibility (Li *et al*., 2022) [14–18]. However, only a few studies have observed the effect from the perspective of a capital market. In the current study, we focus on the effect of the mixed ownership reform on the stock price synchronicity of firms. This effect has not yet been investigated in previous research. Stock price synchronicity reflects the speed and degree of incorporating idiosyncratic information into stock price. It has become an ideal indicator of the information transforming efficiency and resource allocation efficiency of a capital market. Excessive stock price synchronicity not only reduces the efficiency of resource allocation in a capital market and magnifies systemic financial risks, but it also undermines investors’ beliefs on the stock market (Gul *et al*., 2010) [19]. We investigate the following questions in this study: Does the mixed ownership reform improve the information disclosure of firms, and consequently, decrease stock price synchronicity? How does this effect work? Does this effect exhibit any heterogeneity?

China is an ideal place for conducting this research because of two reasons. On the one hand, the mixed ownership reform of SOEs demonstrates strong Chinese characteristics. In contrast with the direct privatisation path of other emerging countries, the mixed ownership reform exhibits the attributes of a public–private dual system, which reflects the basic characteristics of a socialist market economy and embodies the superiority of the Chinese model. Therefore, China is an ideal place for investigating the governance effect of the mixed ownership reform. On the other hand, the institution of law, regulatory enforcement and compliance are weak in the Chinese capital market. Under such circumstance, information disclosure quality in the Chinese capital market is relatively low, and the high synchronicity of stock price is prominent in the Chinese capital market, particularly amongst SOEs (Gul *et al*., 2010) [19]. Thus, China provides an avenue for examining whether the mixed ownership reform exerts a governance effect on the information disclosure of firms.

We find significant evidence that the mixed ownership reform restrains stock price synchronicity. Our mediation analysis further implies that the mixed ownership reform restrains the stock price synchronicity of firms by enhancing executive incentive, strengthening checks and balances amongst shareholders and reducing administration constraints in SOEs. In addition, we perform a cross-sectional analysis and determine that the power of the chief executive officer (CEO), the separation of control and cash flow rights and marketisation degree affect the governance effect of the mixed ownership reform on stock price synchronicity.

The current study contributes to the prior literature in at least two ways. On the one hand, this work enriches relevant research on factors that influence stock price synchronicity. Existing studies have focused on factors at the company level, whilst only a few studies have paid attention to the effect of the mixed ownership reform on a capital market (Gul *et al*., 2010; Ferreira and Laux, 2007; Piotroski and Roulstone, 2004; Chan and Hameed, 2006; Crawford *et al*., 2012) [19–23]. The current study provides systematic evidence on how the mixed ownership reform in SOEs affects the stock price synchronicity of firms, and thus, it offers fresh evidence to fill the gap in the literature. On the other hand, our work improves the understanding of how the mixed ownership reform suppresses opportunistic behaviour in the information disclosure of firms. We identify three mechanisms, namely, the executive incentive, check-and-balance and de-administration mechanisms. Therefore, our study adds to the literature on the governance mechanism of the mixed ownership reform.

The remainder of this paper is organised as follows. Section 2 is the ‘Literature Review’. Section 3 is the ‘Theoretical framework and research hypothesis development’. Section 4 is the ‘Model specification and variable definition.’ Section 5 is the ‘Sample and descriptive statistics’. Section 6 is the ‘Empirical results’, which presents the empirical results and explores possible mechanisms. Section 7 is the ‘Conclusions and policy implications,’ which concludes the obtained results and offers further suggestions on future policymaking.

## 2. Literature review

Two strands of literature are most related to the current study. The first strand discusses the effect of the privatisation of SOEs on their corporate governance, and the effect of different types of property rights on corporate governance.

Previous studies have investigated the relation between the privatisation of SOEs and corporate governance. Some scholars have found that privatisation promotes shareholders to improve the supervision and incentive of managers, reduces management opportunism and moral hazards, and consequently, improves corporate governance (Megginson and Netter, 2001; Gupta, 2005) [24,25]. Cuevas-Rodriguez *et al*. (2016) found that the privatisation of SOEs will strengthen the supervision and incentive of shareholders on management, making management work diligently, and improving the level of corporate governance [26]. Tu and Yu (2015) determined that the privatisation of SOEs can effectively curb the hollowing-out behaviour of major shareholders [27]. Tang’s (2016) research supported this view. An empirical study found that the hollowing-out behaviour of controlling shareholders increases with an increase in the shareholding ratio of state-owned shareholders, whilst non-state-owned shareholders can strengthen checks and balances amongst shareholders, curbing the hollowing-out behaviour of controlling shareholders [28].

Previous studies have also found that the nature of property rights will affect corporate governance (Hassanein *et al*., 2022; Hassanein *et al*., 2023; Geng and Pan, 2024) [29–31]. Borisova and Megginson (2011) reported that compared with private shareholders, state-owned shareholders are more likely to take advantage of implicit guarantee to help enterprises obtain debt financing, reducing financing constraints. Furthermore, the reduction of financing constraints may weaken external supervision [32]. Based on European samples, Borisova *et al*. (2012) determined that state-owned shareholders will harm the corporate governance of firms [33]. Based on an empirical study of 350 privatised companies from 45 countries worldwide, Ben-Nasr *et al*. (2015) found that state-owned shareholders will reduce the quality of earning information [34]. Using Japanese data, Nagata and Nguyen (2017) found that companies with foreign shareholders and domestic institutional investors are more likely to voluntarily and timely provide management earnings forecast revisions [35]. Bennedsen and Wolfenzon (2000) believed that different types of shareholders are conducive to mutual supervision and will promote the improvement of corporate performance [36].

The second literature strand focuses on stock price synchronicity. Stock prices integrate information from the firm, industry and macroeconomic levels (Hassanein *et al*., 2021; Hassanein, 2022; Al-khasawneh *et al*., 2024) [37–39]. Existing studies have found that corporate governance, litigation risk and other related factors will affect corporate information disclosure (Hassanein and Elsayed, 2021; Benameur *et al*., 2023; Elsayed and Hassanein, 2024) [40–42]. Moreover, corporate information disclosure will exert an effect on the profitability and earnings management of firms (Alm El-Din *et al*., 2022; Hassanein and Hussainey, 2015)[43,44]. Stock price synchronicity refers to the correlation between the change in a single company’s stock price and the average change in the market. It is an important indicator for evaluating the efficiency of information disclosure in a capital market (Roll, 1988; Chen *et al*., 2018) [45,46]. Existing research has performed an in-depth analysis of the influencing factors of stock price synchronicity. Gul *et al*. (2010) found that state-owned shareholders will reduce the transparency of corporate information disclosure and improve stock price synchronicity [19]. Based on an empirical study of 654 French listed companies from 1998 to 2007, Boubaker et al. (2014) found that the greater the separation of cash flow and control rights, the higher the stock price synchronicity [47]. Ferreira and Laux (2007) found that the opening of the control market can promote the disclosure of corporate idiosyncratic information and reduce stock price synchronicity [20]. Morck *et al*. (2000) determined that the protection of property rights in a legal system is an important factor that affects stock price synchronicity in a capital market [48]. When the degree of property right protection is weak, the cost of searching for the idiosyncratic information of firms increases for investors, causing stock price synchronicity to increase. Piotroski and Roulstone (2004) determined that the degree of analyst tracking is proportional to stock price synchronicity [21]. This view was also confirmed by Chan and Hameed (2006) [22]. However, Crawford *et al*. (2012) found a nonlinear relationship between analyst tracking degree and stock price synchronicity [23].

## 3. Theoretical framework and research hypothesis development

In this section, we present the theoretical framework for analysis and then posit the hypothesis development.

### 3.1. Theoretical framework

Stock price synchronicity measures the co-movement of individual stock prices with overall market price changes (Roll, 1988) [45]. High stock price synchronicity indicates limited idiosyncratic information disclosure and reduced information transmission efficiency, which are detrimental to firms and the protection of small and medium-sized enterprise investors. The principal–agent problem is determined to be the primary cause of high stock price synchronicity. On the one hand, when the shareholders cannot provide CEOs with a proper compensation incentive, the lack of executive compensation incentive will make executives unwilling to work diligently on information disclosure, reducing the disclosure of idiosyncratic information. On the other hand, large shareholders may take advantage of information to gain the interests of small shareholders, decreasing the disclosure of firms’ idiosyncratic information (Jin and Myers, 2006; Gul *et al*., 2010; Ferreira and Laux, 2007; Piotroski and Roulstone, 2004; Chan and Hameed, 2006; Crawford *et al*., 2012) [19,21–23,49].

Previous studies have found a relatively high stock price synchronicity amongst SOEs, particularly in China (Gul *et al*., 2010; Borisova *et al*., 2012) [19,33]. Compared with private firms, SOEs experience more difficulties in corporate governance. SOEs face the absence of owners, leading to lack of motivation to safeguard national interests, and insufficient incentive and supervision of management. Moreover, the internal administrative governance mechanism of SOEs leads to unreasonable management selection. The phenomenon of the board of directors being ‘in vain’ or the board of directors and the manager being ‘in one’ occurs frequently, weakening the constraints on the behaviour of management and leading to the deterioration of the quality of information disclosure (Borisova *et al*., 2012; Ben-Nasr *et al*., 2015; Apiliyat *et al*., 2024) [5,33,34].

The mixed ownership reform of SOEs offers an opportunity to solve the aforementioned problems. Firstly, by introducing private shareholders into SOEs, the mixed ownership reform solves the absence of owners in SOEs. With a strong profit motive, private shareholders will actively participate in corporate governance, strengthen the supervision and incentive of management and strengthen the supervision of shareholders on management, alleviating agency costs. Secondly, shareholders of different nature are conducive to mutual supervision. They strengthen the check-and-balance effect, preventing major shareholders from taking advantage of information to gain the interests of minority shareholders, and thus, alleviating agency costs in SOEs. Overall, we posit that the mixed ownership reform will decrease agency costs in firms, reducing the stock price synchronicity of firms.

### 3.2. Hypothesis development

In this section, we propose how the mixed ownership reform exerts a governance effect on the information reporting of firms, subsequently influencing the stock price synchronicity of SOEs. We identify three mechanisms, namely, the executive incentive, check-and-balance and de-administration mechanisms.

#### Mechanism 1: Executive incentive mechanism.

The mixed ownership reform will reduce stock price synchronicity by improving executive compensation incentives amongst SOEs. Stock price synchronicity rises when the idiosyncratic information of firms decreases. In such case, executives who are in charge of information disclosure become essential. For our concern, executives with inappropriate compensation incentives will not work diligently in information disclosure, and thus, investors will lack understanding of a company’s idiosyncratic information, further inducing investors to make irrational investment decisions, and aggravating stock price synchronicity.

Previous studies have found that the executive compensation of Chinese SOEs is not commensurate with status and insensitive to corporate performance (Kato and Long, 2006) [50]. Executives in Chinese SOEs have long experienced excessive short-term and insufficient long-term compensation incentives. The imbalance of salary term structure induces the short-sighted behaviour of executives, making them manage market value through selective information reporting disclosure. Moreover, the performance of executives has not been fully compensated due to the ‘executive salary restriction order’, and thus, executives will seek ‘private plots’ beyond their salaries by manipulating firm information disclosure.

The mixed ownership reform can improve the incentive of SOE executives in two ways. On the one hand, the mixed ownership reform will introduce non-state shareholders into SOEs. Compared with state-owned capital, non-state-owned capital is more prone to supervising and motivating executives, reducing the opportunistic behaviour of SOE executives, and thus, improving executive incentive. On the other hand, the mixed ownership reform requires SOEs to establish a modern corporate governance system for the executive incentive. In the traditional corporate governance system of SOEs, executives always fall into contradiction between the objectives of the government and firms, resulting in distorted behaviour in firm information disclosure. Notably, the decision adopted at the Third Plenary Session of the 18th Communist Party of China (CPC) Central Committee pointed out that in the process of deepening the mixed ownership reform, it should ‘establish a professional manager system... reasonably increase the proportion of market-oriented recruitment...’. This condition helps correct the structural imbalance of compensation, and thus, encourages executives to focus on the long-term development of enterprises, strengthen information transmission and actively improve the disclosure level of idiot-specific information.

Overall, the mixed ownership reform can reduce stock price synchronicity through the executive incentive mechanism.

#### Mechanism 2: Check-and-balance mechanism.

The mixed ownership reform can reduce stock price synchronicity by providing effective checks and balances to the board. Existing studies have found that an imbalance of equity governance structure will lead to the failure of supervision mechanisms and create space for executives to seek personal gain (Mork *et al*., 2000) [48]. In such case, controlling shareholders may reduce the disclosure of idiosyncratic information to conceal their opportunistic behaviour, and thus, increasing the stock price synchronicity of firms (Boubaker, 2014) [47]. The introduction of the power to check the largest shareholder in a ‘dominant’ enterprise is a conventional idea for restricting the controlling shareholder’s infringement on other shareholders, particularly on the interests of minority shareholders. The coexistence of multiple major shareholders is frequently regarded as a more effective internal governance mechanism that can reduce the agency costs of a company, preventing controlling shareholders from ‘hollowing out’ (Pagano and Roell, 1998; Attig *et al*., 2008; Boateng and Huang, 2017) [14,51,52].

The mixed ownership reform introduces non-state-owned shareholders into SOEs. In such case, the non-state capital shareholders will exert the check-and-balance effect by improving corporate governance to improve the overall protection of shareholders’ rights and interests (Barroso Casado *et al*., 2016) [53]. Overall, through the check-and-balance mechanism, the mixed ownership reform can eliminate the space for controlling shareholders to seek private gains, weakening their motivation to hide idiosyncratic information, and thus, reducing stock price synchronicity.

#### Mechanism 3: De-administration mechanism.

The mixed ownership reform can reduce the stock price synchronicity of firms by breaking the administration constraints.

Administration management is an important reason for increasing stock price synchronicity in SOEs. On the one hand, executives are mostly appointed by the higher superiors, making executives in SOEs accountable only to higher authorities but also to investors. This situation will lead to the result that executives in SOEs are unwilling to disclose adequately to investors (Wong, 2016) [54]. On the other hand, compared with private enterprises, SOEs have preferential access to scarce resources, such as bank loans, government subsidies, favourable financing and critical natural capital (Wang and Jiang, 2021; Zhou *et al*., 2017) [55,56]. In this context, executives in SOEs do not have an incentive to increase the disclosure of corporate idiosyncratic information to obtain financing.

The mixed ownership reform can break the administrative level of executives in SOEs through the de-administration mechanism, making executives more accountable to minority shareholders rather than to superiors, and thus, executives will be more likely to disclose idiosyncratic information. Moreover, by breaking the soft financing constraint of enterprises through the de-administration mechanism, executives in SOEs are forced to actively release more idiosyncratic information to investors and creditors to decrease the financing cost of enterprises, and thus, reduce the synchronisation of stock prices. Overall, the mixed ownership reform can reduce the synchronicity of stock prices through the de-administration mechanism.

Based on these mechanisms, we propose our hypotheses.

*Hypothesis 1: The mixed ownership reform can decrease the stock price synchronicity of firms.*

## 4. Model specification and variable definition

### 4.1. Model specification

To empirically test the relationship between the mixed ownership reform and the stock price synchronicity of firms, we perform Model (1).


$$Synch_{i,t}=\alpha_{0}+\beta_{1}Mix+\beta_{2}CV+\gamma_{i}+{\text{δ}}_{\text{t}}+{\text{ε}}_{\text{i},\text{t}}$$ (1)

where the dependent variable ${\text{Synch}}_{\text{i},\text{t}}$ refers to the stock price synochronicity of *i* firm at *t* time. The independent variables *Mix* are the equity diversity (*Mixnum*) and equity integration (*Mixrate*). *CV* is a series of control variables, including *Size*, *Lev*, *Roa*, *TonbinQ*, *ROA*, *First*, *Board*, *Dual*, *Msh*, *Indep* and *Big4*. $\gamma_{i}{\text{and δ}}_{\text{t}}$ are vectors of firm and year dummy variables that account for firm and year fixed effects, respectively. Following Petersen (2008), all the regressions in this analysis are standard errors clustered by year and firm [57].

### 4.2. Variable definition

#### 4.2.1. Mixed ownership reform.

In this study, we use the number and proportion of shares of different property rights in SOEs to measure the degree of the mixed ownership reform. By collecting the top 10 shareholders disclosed in the periodic reports of SOEs, this study determines the property right nature and shareholding ratio of the top 10 shareholders one by one (if the shareholders themselves are listed companies, then they are judged in accordance with the nature property right of their actual controllers). State-owned shareholders are defined as shareholders who are directly controlled by the state through relevant government departments, or shareholders formed through state-controlled industrial companies and government-owned investment management companies. A natural person or family shareholder is defined as a shareholder formed by an investment made by a natural person or family in China. Foreign shareholders are defined as shareholders formed by legal enterprises established overseas (including legal enterprises in the Hong Kong, Macao and Taiwan regions and foreign legal enterprises) or overseas natural persons who hold shares of listed companies in China in accordance with relevant law. Private shareholders are defined as shareholders formed by an investment of private enterprises who are nonpublic legal persons in China. Institutional investors are defined as shareholders formed by legal institutions engaged in securities investment in the financial market, including listed open funds (LOFs), qualified foreign investors (QFIs), insurance, fund and securities companies and financial institutions. Other types of shareholders include shareholders other than those of the aforementioned five types, including research institutes, institutions of higher learning, firms, collectives and nonprofit organisations.

Based on clarifying the top 10 shareholders, this study constructs the following indicators to measure the mixed ownership reform of SEOs. (1) Equity diversity (*Mixnum*), defined as the types of different equity properties involved in the top 10 shareholders of an enterprise. If the shareholders involve only one equity nature, then the value of *Mixnum* is 1, the value of two types is 2, and so on. The more types of equity owned by enterprises, the higher the diversity of corporate mixed reform. (2) Equity integration (*Mixrate*), the calculation method of *Mixrate* is as follows: the proportion of state-owned and non-state-owned shares (the sum of the shareholding proportion of other shareholders except for state-owned shareholders) in the total equity of state-owned enterprises defined as *Es* and *Ep*, respectively. The larger *Es* and *Ep* is set as the denominator and the smaller is set as the numerator. The ratio is defined as the *Mixrate*. The higher the ratio, the higher the equity integration between state-owned and non-state-owned capital.

#### 4.2.2. Stock price synchronicity.

Following Piotroski and Roulstone (2004), the weekly return data of stock *i* is regressed [21]. The specific formula for calculating price synchronicity (*Synch*) is as follows:


$$R_{i,w,t}=\beta_{0}+\beta_{1}R_{M,w,t}+\beta_{2}R_{M,w-1,t}+\beta_{3}R_{1,w,t}+\beta_{4}R_{1,w-1,t}+\varepsilon_{i,w,t}$$ (2)

where $R_{i,w,t}$ is the return on investment of company stock *i* in week *w* of year *t,* considering cash dividends. ${\text{R}}_{\text{M},\text{w},\text{t}}$ is the weighted average return on the current market value of all A-share companies in week *w* of year *t*. $R_{1,w,t}$ is the weighted average yield rate of the company’s stock *i* after excluding stock *i* in the industry in week *w* of year *t*. Industry classification in this study is based on the classification standard of China Securities Regulatory Commission in 2012. *R*^*2*^ is calculated, representing the part of stock *i* that is explained by market factors in stock price fluctuation in year *t*. (*1 − R*^*2*^) represents the portion of stock price movements explained by firm-level idiosyncratic information.

We then obtain the price synchronicity of company stock i in year t ($Synch_{i,t}$).


$$Synch_{i,t}=\text{ln}\left[R_{i,t}^{2}/\left(1-R_{i,t}^{2}\right)\right]$$ (3)

#### 4.3.3. Other control variables.

In addition, we control for the factors that may affect the level of stock price synchronicity. All variables are defined in Table 1.

**Table 1:** Definition of variables.

Variable	Definition
*Synch*	The calculation is shown in Formula (3).
*Mixnum*	The types of different equity properties involved in the top 10 shareholders of an enterprise: if the shareholder involves only 1 equity nature, the value of Mixnum is 1, the value of two types is 2 and so on.
*Mixrate*	The proportions of state-owned and non-state-owned shares (the sum of the shareholding proportion of shareholders other than the state-owned shareholders) in all the shares of SOEs are calculated as *Es* and *Ep*, respectively. The larger *Es* and *Ep* are taken as the denominator, whist the smaller ones are taken as the numerator.
*Lev*	Annual average total liabilities/total assets for year *t*
*Size*	Natural logarithm of a company’s total assets at the end of year *t*
*TobinQ*	Market/book value of a company’s shares at the end of year *t*
*Msh*	Ratio of the number of shares held by executives to the total number of shares at the end of year *t*
*Indep*	Proportion of independent directors on the board of directors at the end of year *t*
*Broad*	Number of board members at the end of year *t*
*Dual*	The value is 1 if the chairman and the general manager of a company in year *t* are held by the same person; otherwise, it is 0.
*First*	Proportion of a company’s largest shareholder at the end of year *t*
*Big4*	Dummy variable, whether a company employs one of the Big Four accounting firms, namely, PWC, Deloitte (DTT), KPMG and EY. The value is 1 if yes, and 0 otherwise.
*ROA*	Return on assets, net profit/average total assets

## 5. Sample and descriptive statistics

### 5.1. Sample selection and data sources

Our sample includes SOEs in China’s A-share market during the period of 2006–2020. We hand-extracted the data on ownership categories and the ratios of the top 10 shareholders from the China Stock Market and Accounting Research (CSMAR) database. To accurately define the SOE sample, we collected the ultimate controller’s information from the China Centre for Economic Research (CCER) database. We dropped all the financial companies because these companies typically have special accounting treatment and disclosure requirements in their financial statements. To ensure the accuracy and reliability of the study results, we excluded these companies to improve the consistency and comparability of the data.

### 5.2. Descriptive statistics

The descriptive statistical results of the samples are provided in Table 2. As indicated in the descriptive statistical results, the mean value of stock price synchronicity is 0.490, whilst the standard deviation is 0.2, indicating considerable differences in stock price synchronicity amongst the sample companies. The mean value of *Mixnum* is 3.64, whilst the mean value of *Mixrate* is 0.26, indicating that the integration between state-owned and non-state-owned shareholders is still at a low level. The average leverage ratio (*Lev*) is 0.49. For the control variables, the largest shareholder’s shareholding ratio (*First*) is 38.77%, and the average value of *Dual* is 0.146. The proportion of independent directors (*Indep*) is 37.37%, which exceeds the lower line of 1/3 of the independent directors in listed companies. This study also conducted a Pearson correlation coefficient test, and the absolute values of the correlation coefficients between explanatory and control variables were less than 0.5, indicating less interference of the multicollinearity problem.

**Table 2:** Descriptive statistics.

Variable	N	Mean	SD	Min	P50	Max
*Synch*	9761	0.49	0.20	0.06	0.50	0.92
*Mixnum*	9761	3.64	0.83	2	4	5
*Mixrate*	9761	0.26	0.26	0.01	0.15	1
*Size*	9761	22.88	1.58	19.01	22.67	28
*Lev*	9761	0.49	0.77	0	0.23	4.69
*TobinQ*	9761	1.94	1.43	0.54	1.47	9.71
*ROA*	9761	0.03	0.05	−0.20	0.03	0.21
*First*	9761	38.77%	15.44%	4.42%	37.20%	75.84%
*Board*	9761	2.13	0.19	1.61	2.20	2.71
*Dual*	9761	0.11	0.31	0	0	1
*Msh*	9761	0.86	4.75	0	0	53.82
*Indep*	9761	37.37%	5.97%	16.67%	35.71%	80%
*Big4*	9761	0.11	0.31	0	0	1

This table contains a summary of the statistics of the key variables used in our primary analysis. In particular, it includes the mean, median, standard deviation (SD), minimum (Min), 50% percentile (P50), 75% percentile (P75) and maximum (Max) distributions. The baseline regression sample includes 9761 firm–year observations from 2006 to 2020. All the variables are defined in detail in Table 1.

## 6. Empirical results

### 6.1. Baseline regression

The baseline regression results are provided in Table 3. Columns (1) and (2) show the regression coefficient of equity diversity (Mixnum) and equity integration (Mixrate), respectively. The results in Column (1) show that the coefficient of equity diversity (Mixnum) is − 0.012, which is significant at the 1% level, indicating that the equity diversity of the mixed ownership reform significantly reduces the stock price synchronicity of listed companies. The results in Column (2) show that the coefficient of equity integration (Mixrate) is − 0.077, which is significant at the 1% level, indicating that the integration degree of state-owned and non-state-owned equities in the mixed ownership reform significantly reduces the stock price synchronicity of listed companies. The empirical results indicate that the improvement of the breadth (equity diversity) and depth (equity integration degree) of the mixed ownership reform reduces the synchronicity of the stock prices of SOEs; that is, the mixed ownership reform can effectively promote the disclosure of a company’s characteristic information, eliminating stock price synchronicity in firms.

**Table 3:** Baseline regression.

	(1)	(2)
	*Synch*	*Synch*
*Mixnum*	−0.012***	
	(−4.34)	
*Mixrate*		−0.077***
		(−3.84)
*Size*	0.018***	0.019***
	(3.57)	(3.77)
*Lev*	0.000	−0.001
	(0.02)	(-0.19)
*TobinQ*	−0.014***	−0.012***
	(−4.86)	(−4.16)
*ROA*	−0.003	0.002
	(−0.06)	(0.04)
*First*	−0.000	−0.001
	(−0.89)	(−1.63)
*Board*	−0.021	−0.017
	(−1.07)	(−0.91)
*Dual*	−0.009*	−0.009*
	(−1.69)	(−1.76)
*Msh*	0.001	0.002**
	(1.63)	(2.16)
*Indep*	−0.000	−0.000
	(−0.50)	(−0.47)
*Big4*	0.029**	0.027*
	(1.97)	(1.87)
*Year*	Yes	Yes
*Firm*	Yes	Yes
*_cons*	0.239*	0.200
	(1.90)	(1.62)
*N*	9761	9761
*R* ^*2*^	0.219	0.220

Note: T values based on robust standard errors are enclosed in brackets.

***, ** and *  respectively represent significant levels at 1%, 5% and 10%.

This table presents the results of the effect of the mixed ownership reform on the stock price synchronicity of firms. The independent variables are equity diversity (*Mixnum*) and equity integration (*Mixrate*). The control variables include *Size*, *Lev*, *Roa*, *TonbinQ*, *ROA*, *First*, *Board*, *Dual*, *Msh*, *Indep* and *Big4*. All the variables are defined in detail in Table 1. We also control for the fixed effects of firm and year in the model. The values in parentheses are the T-statistics that correspond to the robust standard errors of clustering at the firm level. ***, ** and *  denote significance levels at 1%, 5% and 10%, respectively.

### 6.2. Endogeneity bias

#### 6.2.1. Two-stage least squares regression.

To address the issue of missing variables, we employ a two-stage least squares approach. Following Kusnadi *et al*. (2015), we use the annual industry average of *Mixnum* and *Mixrate*, excluding their own annual values, namely, *Indnum* and *Indrate,* respectively, as their instrument variables, and then perform two-stage least squares (2SLS) estimation [58].

The regression results are presented in Table 4. Columns (1) and (3) report the regression results of the first stage. The F-values of the first stage in both column are greater than 10, and thus, the weak instrumental variable problem is excluded, indicating that the instrumental variables adopted in this study are effective. Columns (2) and (4) report the regression results of the second stage. The results in Columns (2) and (4) show that the estimated regression coefficients of the main explanatory variables do not change substantially compared with the results of the baseline regression, ensuring the robustness of the conclusions of the baseline regression.

**Table 4:** 2SLS regression.

	(1)	(2)	(3)	(4)
	*Mixnum*	*Synch*	*Mixrate*	*Synch*
*Indnum*	0.987***			
	(35.30)			
*Mixnum*		−0.084***		
		(−9.79)		
*Indrate*			0.723***	
			(39.53)	
*Mixrate*				−0.118***
				(−4.38)
*Control*	yes	yes	yes	yes
*Firm&Year*	yes	yes	yes	yes
*_cons*	0.578***	−0.159***	0.116**	−0.498***
	(2.72)	(−2.71)	(2.43)	(−11.29)
*F*	121.04***		607.92***	
*N*	9761	9761	9761	9761
*R* ^*2*^		0.072		0.144

Note: T values based on robust standard errors are enclosed in brackets.

***, ** and *  respectively represent significant levels at 1%, 5% and 10%.

This table presents the results of the effect of the mixed ownership reform on the stock price synchronicity of firms by using a 2SLS approach. We employ the annual industry average of *Mixnum* and *Mixrate*, excluding their own annual values, namely, *Indnum* and *Indrate*, respectively, as their instrument variables. Columns (1) and (3) provide the first-stage regression results, whilst Columns (2) and (4) present the second-stage regression results. The control variables include Size, Lev, Roa, TonbinQ, ROA, First, Board, Dual, Msh, Indep, and Big4. All the variables are defined in detail in Table 1. We also control for the fixed effects of firm and year in the model. The values in parentheses are the T-statistics that correspond to the robust standard errors of clustering at the firm level. ***, ** and *  denote significance levels at 1%, 5% and 10%, respectively.

#### 6.2.2. Difference-in-difference (DID) and propensity score matching (PSM).

Another endogeneity problem in the baseline results is the sample selection problem. The mixed ownership reform of SOEs is led by the government, and thus, the decision and the degree to join the mixed ownership reform may be affected by the government, resulting in sample selection bias. Therefore, this study adopts the PSM–DID method to solve the endogeneity problem caused by sample selection bias.

We take the transfer of the ultimate controller from the state-owned to the non-state-owned capital as a quasi-natural experiment to employ the DID method. Some SOEs in the sample experience control transfer. That is, the ultimate controller or the largest shareholder of an SEO has changed to a non-state-owned legal person (or natural person). In this study, we set the enterprises with the transfer of the ultimate controller as the experimental group, whilst the enterprises without the transfer of the ultimate controller was set as the control group. Combined with the time of transfer of the control right, we build the DID model. To reduce the difference between the experimental and control groups, the nearest one-to-one PSM was also performed on the samples.

**Fig 1:**
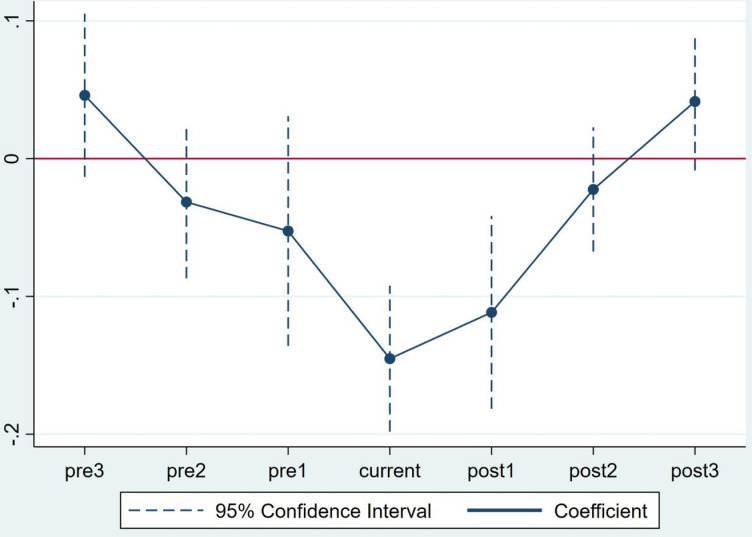
Parallel trend test. Fig 1 shows the results of the parallel test. The figure shows the coefficient of *time×treat,* refers to the experimental group which state-owned enterprises engaged in the mixed ownership reform. According to Fig 1, the coefficients before the mixed ownership reform were insignificant, indicating that no significant difference exists in stock price synchronicity between the experimental and control groups before the mixed ownership reform. Meanwhile, the *time×treat* coefficient decreased significantly after the mixed ownership reform, indicating that the mixed ownership reform significantly decreased firms’ stock price synchronicity in the experimental group.

The premise for an effective use of the DID model is that a parallel trend exists between the experimental and control groups in the sample. In our case, if no control transfer occurs, then the changing trend of stock price synchronicity should be similar between the experimental and control groups. A noteworthy problem is that if the stock price synchronicity of the experimental and control groups exhibits different change trends before the control right transfer, then DID estimation will be invalid. Therefore, a parallel trend test is required. We conduct a regression analysis by adding the interaction terms of the experimental group and the year dummy variables and then judge in accordance with the significance of the regression coefficient and the changing trend of the interaction terms. To visually display the results of the parallel trend test, we draw a trend chart of the regression coefficients of interaction terms (Fig 1). As shown in the figure, most of the *time×treat* coefficients before the transfer of the ultimate control are insignificant, indicating that no significant difference exists in stock price synchronicity between the experimental and control groups before the control right transfer, whilst the interaction term coefficient decreases significantly after control right transfer.

The regression results of PSM–DID are presented in Table 5. In this table, Column (1) shows the estimation coefficients of Mixnum, whilst Column (2) shows the estimation coefficients of Mixrate. In accordance with the results in Columns (1) and (2), equity diversity (Mixnum) and equity integration (Mixrate) receive a significant and negative coefficient, indicating that the mixed ownership reform can significantly reduce stock price synchronicity. Column (3) presents the regression results of PSM–DID. The coefficient estimation result of the interaction term *time×treat* is significantly negative, indicating that the transfer of control rights (from SOEs to non-SOEs) reduces stock price synchronicity. This result guarantees the robustness of the baseline regression results.

**Table 5:** PSM–DID regression.

	*(1)*	*(2)*	*(3)*
	*Synch*	*Synch*	*Synch*
*Mixnum*	−0.017***		
	(−4.31)		
*Mixrate*		−0.104***	
		(−2.66)	
*time×treat*			−0.232***
			(−11.39)
*_cons*	0.723***	0.673***	0.742***
	(3.32)	(3.14)	(3.57)
*Control*	Yes	Yes	Yes
*Firm&Year*	Yes	Yes	Yes
*N*	3724	3681	3730
*R* ^*2*^	0.208	0.207	0.273

Note: T values based on robust standard errors are enclosed in brackets.

***, ** and *  respectively represent significant levels at 1%, 5% and 10%.

This table presents the PSM–DID regression results of the effect of the mixed ownership reform on the stock price synchronicity of firms. We use the enterprises with the transfer of control right as the experimental group, whilst the enterprises without the transfer of control right are used as the control group. The control variables include *Size*, *Lev*, *Roa*, *TonbinQ*, *ROA*, *First*, *Board*, *Dual*, *Msh*, *Indep* and *Big4*. All the variables are defined in detail in Table 1. We also control for the fixed effects of firm and year in the model. The values in parentheses are the T-statistics that correspond to the robust standard errors of clustering at the firm level. ***, ** and *  denote significance levels at 1%, 5% and 10% respectively.

### 6.3. Other robustness checks

We have noted that including industry returns in calculating stock price synchronicity can make separating the industry effect from the market effect difficult (Chan and Hameed, 2006) [22]. To deal with this problem, we follow the research of Chan and Hammed (2006) and take alternative measurements of stock price synchronicity [22]. We exclude industry returns in calculating stock price synchronicity and obtain *Synch2* as an alternative dependent variable to run a robustness test. The results are presented in Table 6. As indicated in this table, equity diversity (*Mixnum*) and equity integration (*Mixrate*) receive a significant and negative effect. This result ensures the robustness of the benchmark regression results.

**Table 6:** Tests for the dependent variables replaced by *Syhch2.*

	(1)	(2)	(3)
	*Synch2*	*Synch2*	*Synch2*
*Mixnum*	−0.012***		
	(−4.29)		
*Mixrate*		−0.072***	
		(−3.64)	
*time×treat*			−0.232***
			(−11.68)
*_cons*	0.245*	0.202	0.773***
	(1.96)	(1.65)	(3.70)
*Control*	Yes	Yes	Yes
*Firm&Year*	Yes	Yes	Yes
*N*	9761	9761	3730
*R* ^*2*^	0.220	0.221	0.272

Note: T values based on robust standard errors are enclosed in brackets.

***, ** and *  respectively represent significant levels at 1%, 5% and 10%.

This table presents the results of the effect of the mixed ownership reform on the stock price synchronicity of firms (*Sych2*). The independent variables are equity diversity *(Mixnum*), equity integration (*Mixrate*) and *time×treat*. The dependent variable is *Sych2.* The control variables include *Size*, *Lev*, *Roa*, *TonbinQ*, *ROA*, *First*, *Board*, *Dual*, *Msh*, *Indep* and *Big4*. All the variables are defined in detail in Table 1. We also control for the fixed effects of firm and year in the model. The values in parentheses are the T-statistics that correspond to the robust standard errors of clustering at the firm level. ***, ** and *  denote significance levels at 1%, 5% and 10%, respectively.

### 6.4. Mediation analysis

The theoretical analysis shows that the mixed ownership reform of SOEs can reduce stock price synchronicity in a capital market through three mechanisms: the executive incentive, check-and-balance and de-administration mechanisms. The three mechanisms are tested empirically in the following sections.

#### 6.4.1. Testing the executive incentive mechanism.

In accordance with the research hypothesis development, the mixed ownership reform will improve the executive incentive, and thus, restrain firm stock price synchronicity. Here, we follow Jensen and Murphy (1990) and establish a model to investigate the effect of the mixed ownership reform on the performance-compensation sensitivity of executives [59]. The model is as follows:


$$�com_{i,t}=\beta_{0}+\beta_{1}Roe_{i,t}+\beta_{2}Mix_{i,t}+\beta_{3}Roe_{i,t}\times Mix_{i,t}+CV+\varepsilon_{i,t},$$ (4)

where the dependent variable *Com* is the executive compensation, and it is calculated as the pair value of the average of the total compensation of the top three executives. The control variables (*CV)* in Formula (4) is the same as those in Formula (1). We also control for the firm and year effects. We expect that the coefficients of interaction of Mix and Roe are positive, such that the mixed ownership reform can strengthen executive incentive. The results are presented in Columns (1) and (2) of Table 7. The coefficients of *Mixnum*×*Roe* and *Mixrate×Roe* are positive and significant, indicating that the mixed ownership reform improves executive incentive, supporting our hypothesis.

**Table 7:** Tests for mediation analysis.

	(1)	(2)	(3)	(4)	(5)	(6)
	*lncom*	*lncom*	*fg*	*fg*	*KZ*	*KZ*
*Roe*	0.038**	0.027**				
	(2.32)	(2.39)				
*Mixnum×Roe*	0.102***					
	(3.21)					
*Mixrate×Roe*		0.203***				
		(3.62)				
*Mixnum*			0.011***		0.084***	
			(3.19)		(4.51)	
*Mixrate*				0.036***		0.041***
				(3.41)		(2.93)
*_cons*	3.359***	3.381***	3.638***	3.510***	−3.282***	−3.223***
	(3.29)	(3.30)	(3.54)	(3.49)	(−11.78)	(−15.37)
*control*	Yes	Yes	Yes	Yes	Yes	Yes
*Firm&Year*	Yes	Yes	Yes	Yes	Yes	Yes
*N*	9,761	9,761	9,761	9,761	9,761	9,761
*R* ^*2*^	0.116	0.115	0.139	0.141	0.426	0.402

Note: T values based on robust standard errors are enclosed in brackets.

***, ** and *  respectively represent significant levels at 1%, 5% and 10%.

This table presents the results of the mediation analysis. Columns (1) and (2) present the effect of the mixed ownership reform on the executive incentive of firms. Columns (3) and (4) present the effect of the mixed ownership reform on the check-and-balance condition of firms. Columns (5) and (6) present the effect of the mixed ownership reform on the financial constraints of firms. The control variables include *Size*, *Lev*, *Roa*, *TonbinQ*, *ROA*, *First*, *Board*, *Dual*, *Msh*, *Indep* and *Big4*. All the variables are defined in detail in Table 1. We also control for the fixed effects of firm and year in the model. The values in parentheses are the T-statistics that correspond to the robust standard errors of clustering at the firm level. ***, ** and *  denote significance levels at 1%, 5% and 10% respectively.

#### 6.4.2. Testing the check-and-balance mechanism.

To investigate whether the mixed ownership reform can improve the check-and-balance mechanism in corporate governance, we must construct a variable for measuring the condition of checks and balances in a firm. We use an excess number of directors appointed by non-state-owned shareholders, namely, *fg*, to measure the check-and-balance conditions of firms. *fg* is calculated as follows. Firstly, the number of appropriately appointed non-state-owned directors is calculated in accordance with the shareholding ratio of non-state-owned shareholders, and then an appropriate number of appointed directors is obtained after the fluctuation of the theoretical number. Finally, the number of over-appointed non-state-owned directors is calculated accordingly.

The results are presented in Columns (3) and (4) of Table 7. The coefficients of *Mixnum* and *Mixrate* on *fg* are positive and significant, indicating that the mixed ownership reform improves the excess number of appointed non-state-owned directors, enhancing checks and balances in corporate governance.

#### 6.4.3. Testing the de-administration mechanism.

In accordance with our hypothesis, the mixed ownership reform can decrease the administration level of SOEs by fastening their financial constraints. In this section, we choose financing constraint (*KZ*) as the intermediary variable to empirically test the de-administration mechanism. Following Kaplan and Zingales (1997), the *KZ* index is calculated as follows: four variables, namely, cash ratio, cash flow ratio (*CF*), asset-to-liability ratio (*Lev*) and dividend payment (*Divdummy*), are selected to measure the financing constraint of a company [60]. We design the following sequential dependent variable, i.e., *KZ1* = 1, for companies with a cash ratio that is lower than the sample median, otherwise *KZ1* = 0; companies with *CF* lower than the sample median *KZ2* = 1, otherwise *KZ2* = 0; companies with *Lev* higher than the sample median *KZ3* = 1, otherwise *KZ3* = 0; and companies that do not pay dividends *KZ4* = 1, otherwise *KZ4* = 0. The dependent variable of the regression equation is the sum of the financing constraints faced by a listed company as *KZ1*–*KZ4* to form a sequence-dependent variable of 0–4. Cash ratio, *CF*, *Lev*, dividend per share and Tobin’s Q are used as independent variables to perform annual and industry-specific regressions of the aforementioned dependent variables. The dependent variables are the ordered data, with five levels from 0 to 4. In the current study, the ordered logistic method is used to perform regression, and the fitting value of the regression equation is the *KZ* index.

We then employ the *KZ* index to investigate the effect of the mixed ownership reform on the financial constraints of firms. The results are presented in Columns (5) and (6) of Table 7.The coefficients of *Mixnum* and *Mixrate* are positive and significant, indicating that the mixed ownership reform has tightened the financing constraints of SOEs, supporting the hypothesis of the de-administration mechanism.

### 6.5. Heterogeneity analysis

The preceding analysis suggests that the mixed ownership reform can exert a corporate governance effect by enhancing executive incentive, improving checks and balances and decreasing the administration level in firms, optimising the information disclosure of firms and restraining their stock price synchronicity. In this section, we further test whether this governance effect varies with changes in CEO power, degree of separation of cash flow and control rights and marketisation degree to investigate the heterogeneity effect.

#### 6.5.1. CEO power.

Executives exert a major influence in information disclosure. Our findings support that the mixed ownership reform in SOEs exerts a positive governance effect by improving executive incentive. In this section, we further test whether CEO power affects the governance effect of the mixed ownership reform. CEO power has been recognised as an important indicator in corporate governance, greater CEO power implies that the CEO has more control over corporate disclosure for personal gain.

Following Coles *et al*. (2014), the current study uses the ‘proportion of new independent directors’ to measure the influence of CEO power on the board of directors [61]. The specific method for calculating CEO power is as follows. (1) The number of new independent directors who join the board of directors after the current CEO takes office is calculated. (2) The influence of the CEO on the board of directors is measured via the proportion of new independent directors amongst the independent directors, i.e., the power of the CEO, which is recorded as Cp.

We then investigate the effect of the mixed ownership reform on stock price synchronicity amongst different levels of CEO power. The results are presented in Table 8. In the table, Columns (1) and (3) list the regression results of the samples with greater CEO power, which is the value of Cp above the sample’ s mean value. Meanwhile, Columns (2) and (4) list the regression results of the samples with less CEO power, which is under the mean value.

**Table 8:** Heterogeneity test of CEO power.

	(1)	(2)	(3)	(4)
	*Synch*	*Synch*	*Synch*	*Synch*
*Mixnum*	−0.105**	−0.027		
	(−2.54)	(−1.65)		
*Mixrate*			−0.188***	−0.031
			(−2.91)	(−0.72)
*Control*	yes	yes	yes	yes
*Firm&Year*	yes	yes	yes	yes
*_cons*	−6.426***	−3.676***	−8.607***	−4.061***
	(−4.44)	(−5.27)	(−4.95)	(−5.32)
*P*	0.003		0.332	
*R* ^*2*^	0.355	0.358	0.333	0.332
*N*	4876	4885	4876	4885

Note: T values based on robust standard errors are enclosed in brackets.

***, ** and *  respectively represent significant levels at 1%, 5% and 10%.

This table presents the results of the heterogeneity effect of the mixed ownership reform on the stock price synchronicity of firms with different levels of CEO power. The independent variables are equity diversity (*Mixnum*) and equity integration (*Mixrate*). Columns (1) and (3) present the regression results of the samples with greater CEO power, whilst Columns (2) and (4) present the regression results of the samples with less CEO power. The dependent variable is *Sych.* The control variables include *Size*, *Lev*, *Roa*, *TonbinQ*, *ROA*, *First*, *Board*, *Dual*, *Msh*, *Indep* and *Big4*. All the variables are defined in detail in Table 1. We also control for the fixed effects of firm and year in the model. The values in parentheses are the T-statistics that correspond to the robust standard errors of clustering at the firm level. ***, ** and *  denote significance levels at 1%, 5% and 10%, respectively.

In accordance with the regression results (1) and (2), the coefficients of Mixnum on stock price synchronicity are both negative but more prominent in the sample with greater CEO power, indicating that the mixed ownership reform can significantly improve the information disclosure efficiency of companies with greater CEO power. In accordance with the regression results in Columns (3) and (4), the coefficients of Mixrate are both negative on stock price synchronicity but more prominent in the samples with greater CEO power, indicating that the mixed ownership reform can significantly improve the information disclosure efficiency of companies with greater CEO power.

#### 6.5.2. Degree of separation of cash flow and control rights.

According to Boubaker *et al*. (2014), the higher the degree of separation of cash flow and control rights, the higher the stock price synchronicity [47]. A higher degree of separation of cash flow and control rights indicates that the interests of managers are not aligned with those of common shareholders. This sections further investigates whether the mixed ownership reform can exert a governance effect to narrow down this difference.

The results are presented in Table 9. Columns (1) and (3) list the regression results of the samples wherein cash flow and control rights are separated. Column (2) and (4) provide the regression results of the samples wherein cash flow and control rights are combined.

**Table 9:** Heterogeneity test of the separation of cash flow and control rights.

	(1)	(2)	(3)	(4)
	*Synch*	*Synch*	*Synch*	*Synch*
*Mixnum*	−0.126**	−0.074*		
	(−2.46)	(−1.84)		
*Mixrate*			−0.136**	−0.122
			(−2.19)	(−1.34)
*Control*	yes	yes	yes	yes
*Firm&Year*	yes	yes	yes	yes
*_cons*	−3.949***	−5.263***	−3.089***	−7.897***
	(−3.45)	(−5.51)	(−4.16)	(−5.69)
*P*	0.014		0.007	
*R* ^*2*^	0.380	0.340	0.338	0.324
*N*	4135	5626	4135	5626

Note: T values based on robust standard errors are enclosed in brackets.

***, ** and *  respectively represent significant levels at 1%, 5% and 10%.

This table presents the results of the heterogeneity effect of the mixed ownership reform on the stock price synchronicity of firms with different levels of separation of cash flow and control rights. The independent variables are equity diversity (*Mixnum*) and equity integration (*Mixrate*). Columns (1) and (3) present the regression results of the samples with separated cash flow and control rights. Columns (2) and (4) present the regression results of the samples with combined cash flow and control rights. The dependent variable is *Sych*. The control variables include *Size*, *Lev*, *Roa*, *TonbinQ*, *ROA*, *First*, *Board*, *Dual*, *Msh*, *Indep* and *Big4.* All the variables are defined in detail in Table 1. We also control for the fixed effects of firm and year in the model. The values in parentheses are the T-statistics that correspond to the robust standard errors of clustering at the firm level. ***, ** and *  denote significance levels at 1%, 5% and 10%, respectively.

In accordance with the regression result in Columns (1) and (2), the coefficients of Mixnum on stock price synchronicity are both negative but more prominent in the sample wherein cash flow and control rights are separated. In accordance with the regression results in Columns (3) and (4), the coefficients of *Mixrate* on stock price synchronicity are both negative but more prominent in the samples wherein cash flow and control rights are separated, indicating that the mixed ownership reform can significantly improve the information disclosure efficiency of companies and narrow down the difference between firms with separated and combined cash flow and control rights.

#### 6.5.3. Marketisation degree.

Corporate governance is inherent in the institutional environment. China’s market-oriented reform is still in its initial stage, and significant differences exist in the market environment of various regions. Therefore, we explore whether the inhibiting effect of the mixed ownership reform on stock price synchronicity is affected by the degree of marketisation. In this section, China’s marketisation index compiled by Fan *et al*. (2011) is used as the proxy variable of marketisation degree and recorded as an index [62]. The current uses the median of the index to divide the samples, and the regression results are presented in Table 10.

**Table 10:** Heterogeneity test of marketisation degree.

	(1)	(2)	(3)	(4)
	*Synch*	*Synch*	*Synch*	*Synch*
*Mixnum*	−0.119***	−0.053*		
	(−2.89)	(−1.85)		
*Mixrate*			−0.159**	−0.046
			(−2.12)	(−1.12)
*Control*	yes	yes	yes	yes
*Firm&Year*	yes	yes	yes	yes
*_cons*	−5.958***	−5.703***	−3.550***	−6.347***
	(−5.21)	(−5.19)	(−5.59)	(−5.87)
*P*	0.002		0.003	
*R* ^*2*^	0.325	0.430	0.309	0.404
*N*	4863	4898	4863	4898

Note: T values based on robust standard errors are enclosed in brackets.

***, ** and *  respectively represent significant levels at 1%, 5% and 10%.

This table presents the results of the heterogeneity effect of the mixed ownership reform on the stock price synchronicity of firms with different marketisation degrees. The independent variables are equity diversity (*Mixnum*) and equity integration (*Mixrate*). Columns (1) and (3) present the regression results of the samples with the two rights separated. Columns (2) and (4) present the regression results of the samples with the two rights combined. The dependent variable is *Sych*. The control variables include *Size*, *Lev*, *Roa*, *TonbinQ*, *ROA*, *First*, *Board*, *Dual*, *Msh*, *Indep* and *Big4*. All the variables are defined in detail in Table 1. We also control for the fixed effects of firm and year in the model. The values in parentheses are the T-statistics that correspond to the robust standard errors of clustering at the firm level. ***, ** and *  denote significance levels at 1%, 5% and 10%, respectively.

In this table, Columns (1) and (3) list the regression results of the samples with a higher degree of marketisation, whilst Columns (2) and (4) list the regression results of the samples with a lower degree of marketisation. In accordance with the regression result in Columns (1) and (2), the coefficient of Mixnum on stock price synchronicity is more prominent in the samples with higher marketisation degree. In accordance with the regression results in Columns (3) and (4), the coefficient of Mixrate on stock price synchronicity is more prominent in the samples with higher marketisation degree. Thus, we can conclude that the governance effect of the mixed ownership reform on stock price synchronicity is more prominent in the samples with higher marketisation degree.

## 7. Conclusions and policy implications

This study offers valuable insights into the governance effect of the mixed ownership reform on SOEs, particularly with regard to information disclosure efficiency in the stock market. The empirical findings support several key conclusions.

Firstly, the mixed ownership reform effectively reduces the stock price synchronicity of SOEs. This finding indicates that the reform contributes to enhancing information disclosure efficiency within the capital market. Secondly, the governance effect of the mixed ownership reform is primarily realised through three key mechanisms: the executive incentive, check-and-balance and de-administration mechanisms. These mechanisms collectively optimise corporate governance and facilitate improved information disclosure. Lastly, the effect of the mixed ownership reform on stock price synchronicity varies based on several factors. (1) CEO power. The governance effect of the mixed ownership reform on stock price synchronicity is more prominent in firms with greater CEO power. This result indicates that the mixed ownership reform can alleviate governance defect due to greater CEO power. (2) Separation degree of rights. Firms in which control and cash flow rights are separated benefit more from the mixed ownership reform in terms of reducing stock price synchronicity. This finding suggests that the reform is particularly effective in mitigating principal–agent conflict. (3) Marketisation degree. Firms with a higher degree of marketisation will experience a more pronounced reduction in stock price synchronicity due to the mixed ownership reform.

Moreover, these findings hold significant theoretical and practical implications. The theoretical implications are as follows. Firstly, this work investigates the economic consequences of the mixed ownership reform of SOEs from the perspective of stock price synchronicity, broadening the research content of the mixed ownership reform of SOEs. By fully considering the special institutional background and corporate governance characteristics of SOEs in China, this study makes a comprehensive analysis of the mechanism and path of the mixed ownership reform of SOEs, which affects the governance effect of information disclosure, providing a useful reference for future studies. Secondly, this study enriches the existing research on factors that affect stock price synchronicity. In contrast with existing research, the current work investigates the effect of the mixed ownership reform on stock price synchronicity, providing new ideas for reducing stock price synchronicity and improving the efficiency of capital market operation. The current research offers a new idea for the governance of information disclosure in a capital market. Existing research has focused on a new regulation issued by regulators or on companies. The current study determines that as a system in the field of corporate governance, the mixed ownership reform of SOEs can essentially improve the environment of capital market information disclosure.

Moreover, this study provides practical implications to policy makers and SOEs. For policy makers, governments should actively explore ways to de-administrate SOEs. Excessive administrative intervention will lead to prominent agency problems in SOEs. On the one hand, the policy burden of enterprises should be eliminated, enabling SOEs to focus on their business goal and improve operating efficiency. On the other hand, the government should reduce the inclination of resources to SOEs, breaking the soft budget constraints on and excessive government subsidies for SOEs, and thus, activating the external governance mechanism of SOEs. For SOEs, corporate governance in SOEs should be actively improved. Enterprises should establish an effective supervision mechanism, implement the selection and appointment of senior executives, strengthen supervision and incentives for managers and reduce executive fraud. In particular, SOEs should appoint non-state shareholders as directors and increase the number of non-state shareholder seats on the board of directors during the mixed ownership reform. Consequently, the check-and-balance mechanism will be strengthened, the construction of modern enterprise mechanisms, such as a market-oriented operation mechanism, will be promoted through mixed property rights and the governance effect of the mixed ownership reform of SOEs will be optimised.

In conclusion, this study provides empirical evidence that the mixed ownership reform is an effective tool for improving corporate governance and reducing stock price synchronicity in SOEs. Recognising the multifaceted effects of the mixed ownership reform and tailoring strategies to specific circumstances can maximise its effectiveness in enhancing information disclosure and overall governance. We also shed light onto further reform in the Chinese capital market. Our study recognises the importance of the mixed ownership reform of SOEs in promoting capital market efficiency, and thus, policy makers should simultaneously promote SOE and capital market reforms, maximising the synergistic effect of two types of reforms.
